# Supplementary material for: Microbial Networks in SPRING - Semi-parametric Rank-Based Correlation and Partial Correlation Estimation for Quantitative Microbiome Data
Source: Front Genet. 2019 Jun 6;10:516. doi: 10.3389/fgene.2019.00516 (PMC6563871; doi:10.3389/fgene.2019.00516)
Supplement: Supplementary file 1 [file Data_Sheet_1.PDF]

frontiers

Supplementary Material

1 INFLUENCE OF SHRINKAGE ON SPR CORRELATION ESTIMATION

The final step in the semi-parametric rank-based (SPR) correlation estimation, put forward in the main manuscript, comprises correlation shrinkage of the form $\widetilde{\Sigma} = (1-\rho)\widehat{\Sigma} + \rho I$ with default value $\rho = 0.01$. This shrinkage step guarantees strict positive definiteness of the final SPR estimator $\widetilde{\Sigma}$. While several theoretically-derived proposals for setting ρ are available in the literature, e.g., Ledoit-Wolf-type shrinkage, we found that, across a wide range of simulations, the overall performance of SPRING is not sensitive to the choice of ρ for small enough ρ ($\rho \ll \sqrt{\log p/n}$). To illustrate this robustness, we show in Figure S1 SPRING's performance in terms of the Hamming distances versus tuning parameter λ for different ρ values for the three graph types considered in the main manuscript. We consider the same simulation setup as in the main manuscript with sample size $n = 500$ and number of taxa $p = 200$. Note that the curves for any $\rho < 0.05$ are nearly identical.

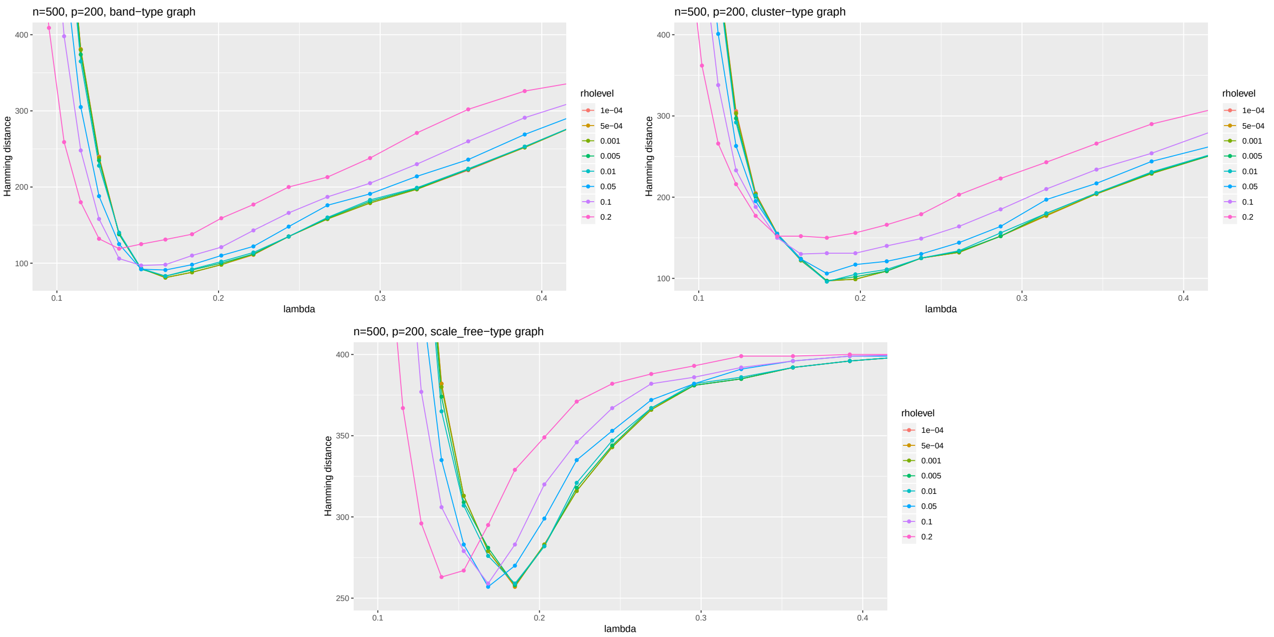

Figure S1. Hamming distances of SPRING-derived association networks to the ground-truth networks as a function of ρ for three graph types. The sample size is $n = 500$ and the number of taxa is $p = 200$.

2 CHOICE OF THE SHIFT PARAMETER C IN THE MCLR$_\varepsilon$ TRANSFORM.

In the main manuscript we introduced the modified centered log ratio transform mclr$_\varepsilon$ for compositional data with the specific purpose of subsequently applying SPR correlation estimation in the presence of relative microbial abundance data.

The mclr$_\varepsilon$ transform comprises the following steps. Let $\mathbf{x}_i \in \mathbb{S}^p$ be the p-dimensional vector of compositions for sample i, and for simplicity of illustration, assume that the first q elements of $\mathbf{x}_i$ are zero, and the other elements are non-zero. We apply

$$\mathbf{z}_i = \mathrm{mclr}_\varepsilon(\mathbf{x}_i) = [0, \ldots, 0, \log\{x_{i(q+1)}/\tilde{g}(\mathbf{x}_i)\} + \varepsilon, \ldots, \log\{x_{ip}/\tilde{g}(\mathbf{x}_i)\} + \varepsilon], \tag{S1}$$

where $\tilde{g}(\mathbf{x}_i) = (\prod_{j=q+1}^{p} x_{ij})^{1/(p-q)}$ is the geometric mean of the non-zero elements of $\mathbf{x}_i$. When $\varepsilon = 0$, mclr$_0$ corresponds to a clr transform applied to non-zero proportions only (Figure 1, middle panel, in the main manuscript). With $\varepsilon > 0$, mclr$_\varepsilon$ applies a positive shift to all non-zero compositions with the purpose of making all non-zero values strictly positive. The ultimate rationale for the shift is to preserve the original ordering of the entries of the compositional vector $\mathbf{x}_i$ (with zeros being the smallest) in the transformed vector $\mathbf{z}_i$. Let $z_{\min} = \min_{ij} \log\{x_{ij}/\tilde{g}(\mathbf{x}_i)\}$. The constraint $\varepsilon > |z_{\min}|$ ensures that $z_{i(q+1)}, \ldots, z_{ip}$ are strictly positive for all i. The default choice in the manuscript is $\varepsilon = |z_{\min}| + c$ with $c = 1$. The fact that SPR-type correlation estimation only considers the ranks of the transformed compositions, implies that the choice of c does not influence the subsequent correlation estimation. However, this may change if other analysis tasks are performed after mclr$_\varepsilon$ transformation of the compositions, most prominently Aitchison-type distance calculations among samples. One alternative way of setting the constant c and thus ε is to connect it to the smallest possible rounding error in the original count data. Specifically, denote the total read count of sample i by T_i. Setting $r_i = 1/(2T_i)$ and calculating $l_i = \log\{r_i/\tilde{g}(\mathbf{x}_i)\}$ leads to $l_{\min} = \min_i l_i$, a global lower bound derived from the smallest possible rounding error. Then, a natural choice for a global shift is to consider $c = (|l_{\min}| - |z_{\min}|)$, leading to $\varepsilon = |l_{\min}|$. Alternatively, a sample-dependent shift $\varepsilon_i = |l_i|$ may also be a valid proposal. We leave the numerical and theoretical evaluation of these choices of the shift for the mclr$_\varepsilon$ for future research.
